# Editorial: Systematic review automation thematic series

**DOI:** 10.1186/s13643-019-0974-z

**Published:** 2019-03-11

**Authors:** Joseph Lau

**Affiliations:** Brown University School of Public Health, Providence, RI 02903 USA

It has been over 35 years since health disciplines adopted systematic reviews and meta-analyses to inform healthcare decisions and policies. Advances in methodology and creation of standards have improved the reliability and consistency of these syntheses of research findings. Conducting a systematic review involves complex processes usually requiring many tedious steps and numerous hours of expert labor. A typical systematic review can take a team of researchers a year or more to produce [1]. Systematic reviews are costly. The cost varies according to the number of questions to answer and the amount of literature to evaluate. For instance, an Agency for Healthcare Research and Quality comparative effectiveness review with five key questions and the need to review about 10,000 citations may cost upward of US$300,000.

Researchers have long used computer technologies to facilitate the production of systematic reviews. They use search engines to conduct searches for potentially eligible studies, use database programs to manage citations, create spreadsheets to collect and manage data, and use statistical software to perform data analyses. Dedicated software has been developed to assist specific aspects of the systematic review process. However, these tools have had minimal impact on the amount of time and costs to produce a systematic review. Had technological innovations of producing systematic reviews paralleled that of microprocessor development, Moore’s law of doubling of computer power every 2 years would imply that a team of researchers today could produce over 260,000 systematic reviews in 1 year using the same amount of resources spent 35 years ago. Looking at it in a different way, ignoring inflation and other cost considerations, hypothetical technological advancements would allow a team today to produce a systematic review for less than a dollar. While these highly exaggerated extrapolations are based on unrealistic assumptions, where we are today, in terms of incorporating technology into the review process, is off by many orders of magnitude. Importantly, systematic reviews today take longer to produce and cost more than 35 years ago.

Given the comprehensive number of healthcare topics requiring formal systematic review and the need to update existing reviews or establish a living systematic review, one can readily see that the demands of research synthesis far outstrip the current supply and capacity of systematic review production, even with new generations of trained reviewers and thousands of Cochrane volunteers, and other groups worldwide. To keep up, the societal costs for review production will reach many tens of millions of dollars (perhaps even more). There is little debate that automation is needed to produce and maintain systematic reviews and to make their availability timely and affordable. Furthermore, the information provided in systematic reviews must be made available in a form that will improve their uptake by the users such as guideline developers and other decision makers. Having automated systematic reviews *talk* to systems that could directly incorporate their results is an opportunity to create a seamless system for evidence-based healthcare decisions and policies.

Over the past 15 years, researchers have begun to develop automation tools for systematic reviews. *Title and abstract screening* is a laborious but essential task in systematic review. This task is one of several bottlenecks in the systematic review process that can take up to several weeks, sometimes months, to complete. It is a low hanging fruit that lends itself to automation using machine learning technology. Some of these tools have advanced far enough to allow them to be used routinely by systematic reviewers supported by the development team. However, the lack of user-friendly software and commercialized products has hindered their widespread adoption. This situation is very likely to change in the near future. *Data extraction* is another labor-intensive task that is a bottleneck in the review process. The potential for increasing efficiency in data extraction through automation is huge. One can envision that automation could generate evidence maps almost at a push of a button. While we are not there yet, we are at the brink of a revolution in automation of systematic reviews, a much-needed development that could spur evidence-based health decision-making and policy.

However, fully automated production of systematic reviews will not happen overnight. Innovation has and will continue to occur in bits and pieces and in myriad forms. In addition, automation will bring challenges; its acceptance is not guaranteed. An obvious central challenge is that of ensuring the trustworthiness of automated systematic reviews. How should we, as a community, evaluate them? Who should attest the accuracy of the findings and products of the automated systematic review? What role should humans play in the conduct of an automated systematic review? Generations of authors have taken academic and other credit from publishing systematic reviews in journals and other venues. Who should take credit for automated systematic reviews? Cheaper and quicker systematic reviews are also no guarantee. In ensuring accuracy, will we create more rigorous and complicated requirements of automated systematic reviews that negate their efficiency gains?

We have commissioned two papers to begin this series on automation of systematic reviews. The paper by Marshall and Wallace provides a brief survey of the state-of-the-science of systematic review automation, discusses some of the challenges and opportunities, and provides some practical guidance on using some of these tools [2]. The second paper by O’Connor and colleagues discusses how innovations in systematic review automation might be evaluated [3]. We hope that these papers will provide food for thought on some of the issues raised in this editorial. We invite authors to submit manuscripts addressing these and other relevant issues (https://www.biomedcentral.com/collections/systrevautomation).

Systematic reviews have revolutionized how evidence is synthesized to inform healthcare decisions. Technologies to automate systematic reviews have simmered in the background for more than a decade. Recent advances in this area have propelled the automation issues into mainstream discussions. For systematic reviews to be successful in the long term, automation is very likely to be part of the process. The community needs to embrace the innovations, address the challenges, and evaluate the effects of automation of the trustworthiness of the product. However, systematic review automation is not a panacea to the challenges of evidence syntheses. Automation alone cannot improve the quality of the evidence. To improve the reliability of the data in systematic reviews, there must be coordinated efforts to improve the quality of information reported in the original publications. The community should also embrace other resources such as clinicaltrials.gov and consider sharing of data extracted from trials included in systematic reviews [4]. By capitalizing existing resources and minimizing unnecessary duplication of effort, we will more likely be able to realize the goals of evidence-based healthcare.
